# The association of body mass index and its interaction with family history of dyslipidemia towards dyslipidemia in patients with type 2 diabetes: a cross-sectional study in Zhejiang Province, China

**DOI:** 10.3389/fpubh.2023.1188212

**Published:** 2023-05-15

**Authors:** Xiang-Yu Chen, Le Fang, Jie Zhang, Jie-Ming Zhong, Jing-Jing Lin, Feng Lu

**Affiliations:** Department of Non-Communicable Disease Control and Prevention, Zhejiang Provincial Center for Disease Control and Prevention, Hangzhou, China

**Keywords:** body mass index, family history of dyslipidemia, interaction, dyslipidemia, diabetes mellitus

## Abstract

**Objectives:**

This study aimed to investigate the association between body mass index (BMI) and dyslipidemia and to explore the interaction between BMI and family history of dyslipidemia towards dyslipidemia in patients with type 2 diabetes.

**Methods:**

This cross-sectional study was conducted between March and November 2018 in Zhejiang Province, China. A total of 1,756 patients with type 2 diabetes were included, physical examination data, fasting blood samples and face-to-face questionnaire survey data were collected. Restricted cubic spline analysis was used to evaluate the association between BMI and the risk of dyslipidemia. Unconditional multivariable logistic regression was used to estimate the interaction between BMI and family history of dyslipidemia towards dyslipidemia.

**Results:**

The prevalence of dyslipidemia was 53.7% in the study population. The risk of dyslipidemia elevated with increased BMI value (*p* for non-linearity <0.05). After adjusting for covariates, individuals with high BMI (≥24 kg/m^2^) and a family history of dyslipidemia had a 4.50-fold (95% CI: 2.99–6.78) increased risk of dyslipidemia compared to the normal reference group, which was higher than the risk associated with high BMI alone (OR = 1.83, 95% CI: 1.47–2.28) or family history of dyslipidemia alone (OR = 1.79 95% CI: 1.14–2.83). Significant additive interaction between high BMI and a family history of dyslipidemia was detected, with RERI, AP, and SI values of 1.88 (95% CI: 0.17–4.10), 0.42 (95% CI: 0.02–0.62), and 2.16 (95% CI: 1.07–4.37), respectively. However, stratified by status of diabetes control, this additive interaction was only find significant among patients with controlled diabetes.

**Conclusion:**

Both high BMI and a family history of dyslipidemia were related with high risk of dyslipidemia. Moreover, there were synergistic interaction between these two factors. Patients with type 2 diabetes who had a family history of dyslipidemia were more susceptible to the negative impact of being overweight or obesity on dyslipidemia.

## Introduction

The latest study has reported that diabetes affects approximately 11% of the Chinese population, and its prevalence is still on the rise, posing a significant public health challenge in China (1). Meanwhile, previous study reported that the prevalence of dyslipidemia was 67.1% among the type 2 diabetes populations (2). The co-occurrence of diabetes and dyslipidemia is of particular concern since it significantly increases the risk of developing cardiovascular disease, which is a leading cause of morbidity and mortality in individuals with diabetes (3–5).

Body mass index (BMI) is a commonly used measure of adiposity, the high level of BMI was indicated to be associated with an increased risk of dyslipidemia in some studies (6–8). In addition to BMI, family history of dyslipidemia is also a known risk factor for developing dyslipidemia (9, 10). Previous research suggested that family history of dyslipidemia plays a significant role in the development of cardiometabolic alterations (9, 11, 12). Despite this, to date, there is limited knowledge on the association between BMI and family history of dyslipidemia in relation to dyslipidemia among individuals with diabetes. Given the high prevalence of dyslipidemia in people with diabetes, to understand the effect measure modification between BMI and family history of dyslipidemia towards dyslipidemia in people with diabetes is important for identifying high-risk individuals and developing effective prevention and treatment strategies.

Thus, the aim of this study is to analyze the characteristics of dyslipidemia in people with diabetes by utilizing data from the Zhejiang Provincial Diabetic Complications Study. Specifically, we seek to investigate the association of BMI and its effect measure modification with family history of dyslipidemia on the risk of dyslipidemia. The findings of this study will provide a basis for the prevention and control of dyslipidemia in the populations with type 2 diabetes.

## Methods

### Study design and population

During the period of March to November 2018, the Zhejiang Provincial Diabetic Complications Study was conducted as part of the China National Diabetic Complications Study. The study aimed to investigate the prevalence and risk factors of diabetic complications among patients with type 2 diabetes in Zhejiang Province, China. A detailed description of the study design, methods, and participants can be found elsewhere (13). In brief, the study recruited eligible participants who were registered patients with type 2 diabetes living in the survey sites for more than 6 months within 12 months before the survey, aged 18 years or older, and were not pregnant, bedridden, or intellectually disabled.

The study adopted a multi-stage random sampling method (13), Firstly, two districts and two counties were randomly selected from Zhejiang Province. Secondly, four streets/towns were randomly selected from each district/county. Thirdly, 120 patients with type 2 diabetes registered in the local health system were randomly selected from each street/town based on gender and age stratification, resulting in a total of 1,920 eligible participants. The study collected physical examination data, fasting blood samples, and conducted a face-to-face detailed questionnaire survey for all participants.

This study was approved by the ethics committee (Approval No: 2018-010) (13), written informed consent was obtained from each participant prior to data collection.

### Data collection

The questionnaire survey collected data on participants’ demographic and socioeconomic information, health-related behaviors, and family history of certain diseases via face-to-face interviews conducted by well-trained staff from the center for disease control and prevention (CDC) or primary health care institutions. Physical measurements and blood sample collection were carried out by trained personnel in primary health care institutions. Demographic and socioeconomic information included gender, age, residential area, and educational level, etc. Health-related behaviors included cigarette smoking, alcohol consumption, tea, and coffee consumption, etc. Family history of certain diseases included hypertension, diabetes, obesity, coronary heart disease, stroke, tumor, and dyslipidemia, etc. Physical examinations involved measuring body height, weight, waist circumference, and blood pressure. Height was measured using a TZG height gage with a precision of 0.1 centimeters (cm), while weight was measured using a TANITA HD-390 body weight scale with a precision of 0.1 kilograms (kg). Blood pressure was measured three times, with a 1-min rest interval, using an Omron HBP-1300 blood pressure monitor. Blood samples were tested for fasting plasma glucose (FPG), glycosylated hemoglobin (HbA1c), uric acid (UA), triglyceride (TG), total cholesterol (TC), low-density lipoprotein-cholesterol (LDL-C), and high-density lipoprotein-cholesterol (HDL-C), etc. The lipids (TC, TG, HDL-C, LDL-C) and UA were measured using enzymatic methods (Roche cobas c701, Switzerland). FPG was measured using the hexokinase method, while HbA1c was measured using the high-performance liquid chromatography method (BioRad D10 Hemoglobin Analyzer, USA).

### Definition of the variables

#### Outcome variable

Dyslipidemia was used as outcome variable. The diagnostic criteria for dyslipidemia were subjects having any one of the following: TC level ≥ 6.22 mmol/L; TG level ≥ 2.26 mmol/L; LDL-C level ≥ 4.14 mmol/L; HDL-C level < 1.04 mmol/L; had been diagnosed as dyslipidemia or hyperlipidemia by clinical institutions (14).

#### Independent variables

In our study, BMI and family history of dyslipidemia were considered as independent variables. BMI was calculated as weight in kilograms divided by the square of height in meters (kg/m^2^). According to the guidelines for prevention and control of overweight and obesity in Chinese adults (15), BMI was divided into low and normal BMI (<24 kg/m^2^) and high BMI (≥24 kg/m^2^). Family history of dyslipidemia was defined as the presence of dyslipidemia in parents or siblings of a patient with type 2 diabetes.

### Covariates

The following categorical variables were considered to be potential covariates adjusted in the multivariate logistic regression model. Age group: according to the prior studies in China, participants in this study were categorized into young adults (≥18 years and < 45 years old), middle-aged adults (≥45 years and <60 years old) and old adults (≥60 years old) groups (16). Gender was categorized as male or female. The education levels were divided into secondary school or lower, senior high school, college or above (17). Residence area was classified as urban or rural based on the participant’s residential location, either in an urban district or a county. Hypertension was defined as systolic blood pressure(SBP) ≥140 mmHg and/or diastolic blood pressure(DBP) ≥90 mmHg and/or a self-reported diagnosis of hypertension (18). Current cigarette smoking was defined as an individual currently smokes cigarette every day or some days. Alcohol drinking was defined as an individual have drunk alcohol within the past 30 days or 30 days ago. The diagnostic criteria for hyperuricemia was defined as uric acid (UA) >420 μmol/L in both males and females. Abnormal FPG and abnormal HbA1c were defined as FPG ≥ 7.0 mmol/L and HbA1c ≥7.0%, respectively (19).

### Statistical analysis

SAS (version 9.4) software was used to analyze the data. Continuous data was described as means with standard deviations (SD) or median (interquartile range, IQR). Differences of continuous data between groups were analyzed using *t*-test or Wilcoxon rank-sum test. Qualitative data was described as frequency (percentage) and chi-square test (*χ*^2^-test) was used to compare the differences between these different groups. Restricted cubic spline model was used to evaluate the association between BMI levels and risk of dyslipidemia. An unconditional multivariable logistic regression model was used to estimate the influencing factors of dyslipidemia, and the backward elimination method was used to select variables for confounder adjustment. The results of this unconditional multivariable logistic regression model and the interaction table were used to analyze the additive interaction between BMI and family history of dyslipidemia (20). The relative excess risk due to interaction (RERI), the attributable proportion due to interaction (AP) and the synergy index (SI) were calculated (21). The table produced by T Anderson was used to calculate the 95% confidence interval (CI) of the interaction indicators (22). If the RERI equal to 0, it means there is no additive interaction, if the RERI greater than 0, it indicates a positive additive interaction (23). If the 95% CI of AP or RERI contains 0 or the 95% CI of SI contains 1, it means that the two factors have no interaction. The level of significance (α) in this study was set at 0.05.

## Results

### Basic characteristics of the subjects

The study enrolled 1,756 out of 1,920 participants who provided complete research information. Among the participants, 876 (49.9%) were males, with a mean age of 57.2 ± 10.2 years and a mean BMI of 24.8 ± 3.4 kg/m^2^. Dyslipidemia was found in 943 (53.7%) subjects, hypertension in 1,147 (65.3%), hyperuricemia in 292 (16.6%), and 260 (14.8%) had a family history of dyslipidemia. Furthermore, 436 (24.8%) were current cigarette smokers, and 646 (36.8%) were alcohol drinkers. Table 1 presents the basic characteristics of the 1,756 subjects.

**Table 1:** Basic characteristics of the subjects (*n* = 1,756).

Characteristics	Participants overall	Subjects without dyslipidemia	Subjects with dyslipidemia	*t*/*χ*^2^/*z*	Value of *p*
Overall *n* (%)	1,756	813 (46.3)	943 (53.7)		
Age (years) [means ± SD]	57.2 ± 10.2	57.6 ± 10.2	56.9 ± 10.1	1.34^a^	0.181
Gender *n* (%)				5.99^b^	0.014
Male	876 (49.9)	380 (46.7)	496 (52.6)		
Female	880 (50.1)	433 (53.3)	447 (47.4)		
Educational level *n* (%)				8.36^b^	0.015
Secondary school and lower	1,541 (87.8)	733 (90.2)	808 (85.7)		
Senior high school	171 (9.7)	65 (8.0)	106 (11.2)		
College or above	44 (2.5)	15 (1.8)	29 (3.1)		
Residence				22.80^b^	<0.001
Rural	875 (49.8)	455 (56.0)	420 (44.5)		
Urban	881 (50.2)	358 (44.0)	523 (55.5)		
BMI (kg/m^2^) [means ± SD]	24.8 ± 3.4	23.9 ± 3.2	25.5 ± 3.4	−10.60^a^	<0.001
SBP (mmHg) [means ± SD]	136.5 ± 18.7	134.4 ± 18.3	138.2 ± 18.9	−4.21^a^	<0.001
DBP (mmHg) [means ± SD]	78.4 ± 10.7	76.8 ± 10.3	79.7 ± 10.8	−5.79^a^	<0.001
TG (mmol/L) [median(IQR)]	1.6 (1.1–2.4)	1.2 (0.9–1.6)	2.3 (1.6–3.3)	−24.32^c^	<0.001
TC (mmol/L) [means ± SD]	4.7 ± 1.1	4.5 ± 0.7	4.8 ± 1.3	−7.05^a^	<0.001
HDL-C (mmol/L) [means ± SD]	1.2 ± 0.4	1.4 ± 0.3	1.1 ± 0.3	23.39^a^	<0.001
LDL-C (mmol/L) [means ± SD]	2.7 ± 0.9	2.7 ± 0.7	2.7 ± 1.1	0.37^a^	0.712
FPG (mmol/L) [means ± SD]	7.9 ± 2.6	7.7 ± 2.4	8.1 ± 2.7	−2.96^a^	0.003
HBA1c (%) [means ± SD]	7.3 ± 1.5	7.2 ± 1.5	7.4 ± 1.5	−2.87^a^	0.004
UA (mmol/L) [means ± SD]	334.6 ± 94.4	315.6 ± 87.3	351.1 ± 97.3	−7.98^a^	<0.001
Family history of dyslipidemia *n* (%)	260 (14.8)	77 (9.5)	183 (19.4)	34.16^b^	<0.001

^a^Student’s *t*-test; ^b^Chi-square test; ^c^Wilcoxon rank-sum test.

BMI, body mass index; SBP, systolic blood pressure; DBP, diastolic blood pressure; TG, triglycerides; TC, total cholesterol; HDL-C, high density lipoprotein-cholesterol; LDL-C, low density lipoprotein-cholesterol; FPG, fasting plasma glucose; UA, uric acid.

### Multivariable analysis on influencing factors of dyslipidemia in patients with type 2 diabetes

The multivariable logistic regression results on influencing factors of dyslipidemia are presented in Table 2. The results indicate that in addition to BMI and family history of dyslipidemia, urban residence (OR = 1.67, 95%CI: 1.36–2.06), hyperuricemia (OR = 1.83, 95%CI: 1.37–2.44), hypertension (OR = 1.91, 95%CI: 1.52–2.39), abnormal HbA1c (OR = 1.42, 95%CI: 1.16–1.73), and current cigarette smoking (OR = 1.57, 95%CI: 1.18–2.08) are significantly associated with an elevated risk of dyslipidemia. After adjusting for these factors, the risk of dyslipidemia in patients with type 2 diabetes with high BMI is 1.92 times higher than that in those with low or normal BMI (95%CI: 1.57–2.37). The risk of dyslipidemia in patients with type 2 diabetes with a family history of dyslipidemia is 2.30 times higher than that in those without a family history of dyslipidemia (95%CI: 1.73–3.06).

**Table 2:** Multivariable analysis on influencing factors of dyslipidemia (*n* = 1,756).

Variables		Subjects without dyslipidemia *N* = 813 N(%)	Subjects with dyslipidemia *N* = 943 N(%)	OR(95%CI)	Value of *p*
Gender	Female	433 (53.3)	447 (47.4)		
	Male	380 (46.7)	496 (52.6)	0.95 (0.74–1.22)	0.688
Age (Years)	18–44	112 (13.8)	151 (16)		
	45–59	354 (43.5)	413 (43.8)	0.83 (0.61–1.12)	0.224
	≥60	347 (42.7)	379 (40.2)	0.71 (0.51–0.98)	0.036
Residence	Rural	455 (56)	420 (44.5)		
	Urban	358 (44)	523 (55.5)	1.67 (1.36–2.06)	<0.001
Family history of dyslipidemia	No	736 (90.5)	760 (80.6)		
	Yes	77 (9.5)	183 (19.4)	2.30 (1.73–3.06)	<0.001
BMI (kg/m^2^)	<24	425 (52.3)	309 (32.8)		
	≥24	388 (47.7)	634 (67.2)	1.92 (1.57–2.37)	<0.001
HUA	No	723 (88.9)	741 (78.6)		
	Yes	90 (11.1)	202 (21.4)	1.83 (1.37–2.44)	<0.001
Hypertension	No	350 (43.1)	259 (27.5)		
	Yes	463 (56.9)	684 (72.5)	1.91 (1.52–2.39)	<0.001
Hba1c abnormal	No	449 (55.2)	448 (47.5)		
	Yes	364 (44.8)	495 (52.5)	1.42 (1.16–1.73)	0.001
Current cigarette smoking	No	641 (78.8)	679 (72)		
	Yes	172 (21.2)	264 (28)	1.57 (1.18–2.08)	0.002
Alcohol drinking	No	527 (64.8)	583 (61.8)		
	Yes	286 (35.2)	360 (38.2)	0.80 (0.64–1.01)	0.062

OR, odds ratio; CI, confidence interval; BMI, body mass index; HUA, hyperuricemia.

### Dose–response relationship between BMI and dyslipidemia

**Figure 1:**
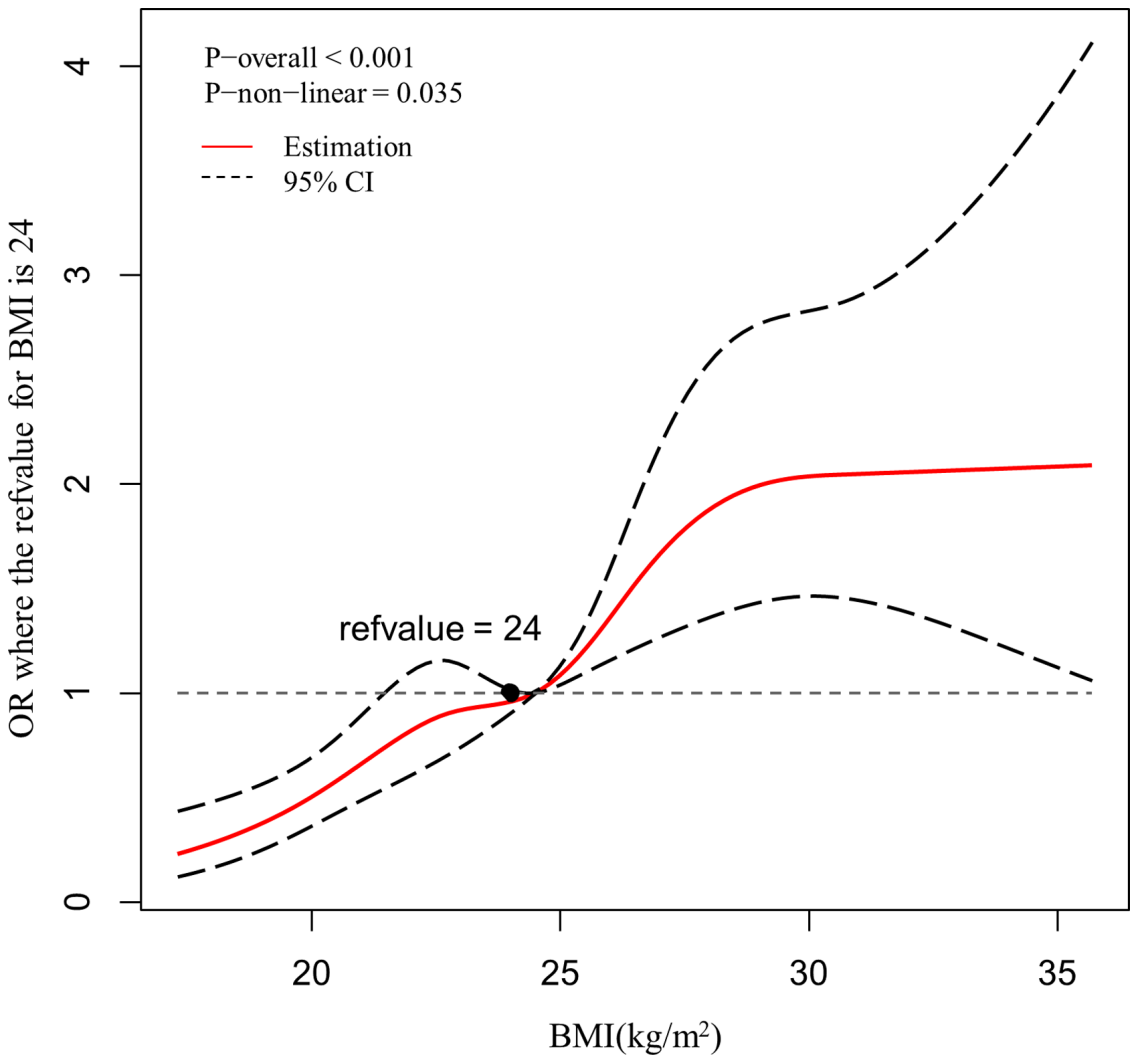
Association between BMI and the risk of dyslipidemia, allowing for nonlinear effects, with 95%CI. The model shows ORs compared with BMI 24  kg/m^2^, adjusting for age, gender, residence, hypertension, hyperuricemia, HbA1c abnormal, current cigarette smoking. BMI, body mass index; CI, confidence interval; OR, odds ratio.

A restricted cubic spline model was used to evaluate and visualize the relationship between BMI and the risk of dyslipidemia (Figure 1). The reference value (OR = 1) was set at BMI = 24 kg/m^2^. The figure shows that there is non-linearity association between BMI and the risk of dyslipidemia (p for non-linearity<0.05). The figure indicates a sustained elevation of the risk with increased BMI, this elevation becomes flat around BMI = 28 kg/m^2^ and larger.

### The interaction between BMI with family history of dyslipidemia towards dyslipidemia

Participants were categorized into four groups based on their BMI levels: ≥24 kg/m^2^ (high BMI), <24 kg/m^2^ (low and normal BMI) and family history of dyslipidemia. Compared to underweight or normal weight subjects without family history of dyslipidemia, patients with high BMI and a family history of dyslipidemia had the highest risk of dyslipidemia (crude OR = 5.30, 95%CI:3.56–7.89; multi-adjusted OR = 4.50, 95%CI:2.99–6.78; Table 3). There was a significant additive interaction between BMI and a family history of dyslipidemia towards dyslipidemia with the RERI, AP, and SI were 1.91 (95% CI: 0.18–4.17), 0.42 (95% CI: 0.02–0.62), and 2.15 (95% CI: 1.07–4.33), respectively (Table 4). More specifically, BMI and a family history of dyslipidemia acted synergistically towards dyslipidemia.

**Table 3:** The interaction analysis on BMI and family history of dyslipidemia towards dyslipidemia (*n* = 1,756).

Variables		Dyslipidemia (*n*, %)	Model 1	Model 2
Family history of dyslipidemia	BMI (kg/m^2^)		OR (95%CI)	OR (95%CI)
Overall				
No	<24	257 (40.0)	1.00 (ref)	1.00 (ref)
	≥24	503 (58.9)	2.15 (1.74–2.65)	1.83(1.47–2.28)
Yes	<24	52 (56.5)	1.95 (1.25–3.03)	1.79(1.14–2.83)
	≥24	131 (78.0)	5.30 (3.56–7.89)	4.50(2.99–6.78)
Controlled diabetes				
No	<24	126 (37.1)	1.00 (ref)	1.00 (ref)
	≥24	219 (53.2)	1.93 (1.44–2.58)	1.68(1.24–2.28)
Yes	<24	33 (55.9)	2.16 (1.23–3.77)	1.95 (1.10–3.45)
	≥24	70 (81.4)	7.43 (4.14–13.35)	6.35 (3.49–11.58)
Uncontrolled diabetes				
No	<24	131 (43.4)	1.00 (ref)	1.00 (ref)
	≥24	284 (64.3)	2.35 (1.74–3.17)	1.94 (1.42–2.31)
Yes	<24	19 (57.6)	1.77 (0.86–3.67)	1.60 (0.78–3.42)
	≥24	61 (74.4)	3.79 (2.20–6.54)	3.20 (1.82–5.63)

BMI, body mass index; OR, odds ratio; CI, confidence interval.

Model 1: unadjusted any covariate; Model 2: adjusted for age, gender, residence, hypertension, hyperuricemia, HbA1c abnormal, current cigarette smoking.

Controlled diabetes: HbA1c < 7.0%; uncontrolled diabetes: HbA1c ≥ 7.0%.

**Table 4:** Indicators of additive interaction between BMI and family history of dyslipidemia (*n* = 1,756).

Overall	Variables		RERI (95%CI)	AP (95%CI)	SI (95%CI)
Model 1	With a family history of dyslipidemia	BMI ≥ 24 kg/m^2^	2.21 (0.29–4.73)	0.42 (0.05–0.61)	2.06 (1.11–3.81)
Model 2	With a family history of dyslipidemia	BMI ≥ 24 kg/m^2^	1.88 (0.17–4.10)	0.42 (0.02–0.62)	2.16 (1.07–4.37)
Controlled diabetes					
Model 1	With a family history of dyslipidemia	BMI ≥ 24 kg/m^2^	4.35 (0.92–10.11)	0.59 (0.15–0.75)	3.09 (1.31–7.30)
Model 2	With a family history of dyslipidemia	BMI ≥ 24 kg/m^2^	3.71 (0.72–8.76)	0.59 (0.12–0.76)	3.29 (1.26–8.60)
Uncontrolled diabetes					
Model 1	With a family history of dyslipidemia	BMI ≥ 24 kg/m^2^	0.67 (−1.76–3.38)	0.18 (−0.68–0.51)	1.32 (0.52–3.32)
Model 2	With a family history of dyslipidemia	BMI ≥ 24 kg/m^2^	0.65 (−1.60–3.04)	0.20 (−0.73–0.54)	1.42 (0.47–4.25)

OR, odds ratio; CI, confidence interval; RERI, relative excess risk due to interaction; AP, attributable proportion due to interaction; SI, synergy index.

Model 1: unadjusted any covariate; Model 2: adjusted for age, gender, residence, hypertension, hyperuricemia, HbA1c abnormal, current cigarette smoking.

We further conducted the analysis of the additive interaction across different status of diabetes control and the inconsistent results was revealed. This additive interaction was significant among patients with controlled diabetes (RERI = 3.71, 95%CI:0.72–8.76; AP = 0.59, 95%CI:0.12–0.76; SI = 3.29, 95%CI:1.26–8.60; Table 4). However, this additive interaction was not significant among patients with uncontrolled diabetes (RERI = 0.65, 95%CI: −1.60-3.04; AP = 0.20, 95%CI: −0.73-0.54; SI = 1.42,95%CI:0.47–4.25; Table 4).

## Discussion

Our study provides the first prevalence data of dyslipidemia among individuals with type 2 diabetes in Zhejiang Province, China. In 2018, the prevalence of dyslipidemia in this population was found to be 53.7%, which is slightly lower than the 59.3% reported in Yaru Li′s study (14), but significantly higher than the national prevalence of 34.0% in the general population of China (24). Given the large number of individuals with type 2 diabetes in Zhejiang Province (25), the high percentage of dyslipidemia in this population represents a significant hidden threat to both social and economic development.

BMI is a widely used measure of body fat based on an individual’s height and weight. Our findings revealed a significant non-linear dose–response relationship between BMI and the risk of dyslipidemia (p for non-linearity<0.05). Our study found evidence of a dose–response relationship between BMI and dyslipidemia among overweight and obesity diabetes patients. This was consistently with some previous studies (6, 26). The mechanism is thought to be related with metabolic effects of excess body fat, which can lead to insulin resistance, inflammation, and some other physiological changes that disrupt the normal lipid metabolism (27).

Our study identified family history of dyslipidemia as an independent risk factor for dyslipidemia, which is consistent with several previous studies (9, 28). While the exact mechanisms by which family history of dyslipidemia increases the risk of dyslipidemia are not fully understood, it is believed that genetic factors may play a role (29, 30). Certain genes have been identified that increase the risk of dyslipidemia, including genes that regulate cholesterol metabolism and lipoprotein synthesis (31). In addition, environmental factors may contribute to the association between family history of dyslipidemia and dyslipidemia. Individuals with family members who have dyslipidemia may live in the environment where unhealthy lifestyle behaviors like consuming a diet high in saturated fats, being physically inactive, or smoking were common due to the cultural/social norm (9).

Our study was the first clue to implicate an additive interaction between BMI and family history of dyslipidemia towards dyslipidemia in patients with type 2 diabetes. Specifically, the effect of BMI on dyslipidemia was significantly modified by family history of dyslipidemia. This finding is noteworthy as there are limited studies investigating this interaction. Despite this, our study was consistent with some similar studies. For example, in a previous study conducted on a population of Filipinos, it was observed that the APOA5 Gly185Cys variant had a significant interaction with waist circumference in relation to triglyceride levels and the effects of the variant on plasma triglycerides became more pronounced as the waist circumference increased (32). Several other studies also had indicated that the genetic susceptibility to hypertriglyceridemia is notably exacerbated by obesity, while the advantageous impact of genetic variants that raise HDL-C is diminished (33, 34). Gene–environment interactions may contribute to this interaction effect in our study (35), whereby genetic variants associated with dyslipidemia may interact with environmental factors (such as high-calorie diets and low physical activity) to increase the risk of dyslipidemia in individuals with a high BMI.

Further stratified by status of diabetes control, we found that this interaction was significant among patients with controlled diabetes. However, it was not significant among patients with uncontrolled diabetes. One possible reason is that the uncontrolled diabetes can lead to hypertriglyceridemia due to high dihydroxyacetone phosphate (DHAP) availability (36). The exact mechanism remains to be further studied.

Our study findings hold significant implications for the prevention and management of dyslipidemia among patients with type 2 diabetes. Specifically, individuals with diabetes who have a family history of dyslipidemia and a high BMI should receive close monitoring for dyslipidemia, with prompt interventions implemented to prevent its development. Even in patients with good glycemic control, this should not be ignored. Since the family history is an unmodifiable risk factor, controlling BMI remains a critical strategy for reducing dyslipidemia risk. Lifestyle modifications, such as dietary changes and physical exercise, may be particularly effective in individuals with a family history of dyslipidemia and a high BMI.

## Limitation

Our study had several limitations that need to be acknowledged. Firstly, the self-reporting of family history of dyslipidemia, current cigarette smoking and alcohol drinking by subjects may have introduced information bias. Secondly, the cross-sectional nature of our study limits our ability to establish causality between BMI, family history of dyslipidemia and dyslipidemia in individuals with type 2 diabetes. Thirdly, our study was conducted in a specific population with type 2 diabetes in China, and may not be generalizable to other populations. Future longitudinal studies using larger, more diverse populations are needed to confirm our findings.

## Conclusion

Our study uncovered a significant synergistic interaction between high BMI and family history of dyslipidemia in relation to dyslipidemia among individuals with diabetes. This finding highlights the need to account for this interaction when evaluating dyslipidemia risk in the populations with type 2 diabetes. Our study can help to identify more beneficial subgroups for dyslipidemia prevention in the case of limited healthcare resources. Our study could provide clues for future corresponding mechanistic studies regarding these two factors.

## Data availability statement

The raw data supporting the conclusions of this article will be made available by the authors, without undue reservation.

## Ethics statement

The studies involving human participants were reviewed and approved by Ethics committee of Shanghai Jiao Tong University Affiliated Sixth People’s Hospital. The patients/participants provided their written informed consent to participate in this study.

## Author contributions

X-YC and LF contributed to conception and design of the study. FL and JZ collected the data. X-YC, JZ, and J-JL organized the database. X-YC performed the statistical analysis. X-YC and J-MZ wrote the first draft of the manuscript. All authors contributed to the article and approved the submitted version.

## Funding

This study was supported by “Zhejiang Provincial Medical Health and Technology Project (2020RC049)” and “Zhejiang Provincial Soft Science Research Program (2022C35013).”

## Conflict of interest

The authors declare that the research was conducted in the absence of any commercial or financial relationships that could be construed as a potential conflict of interest.

## Publisher’s note

All claims expressed in this article are solely those of the authors and do not necessarily represent those of their affiliated organizations, or those of the publisher, the editors and the reviewers. Any product that may be evaluated in this article, or claim that may be made by its manufacturer, is not guaranteed or endorsed by the publisher.
